# Recent Advances in the Study of Wheat Protein and Other Food Components Affecting the Gluten Network and the Properties of Noodles

**DOI:** 10.3390/foods11233824

**Published:** 2022-11-27

**Authors:** Peng Zang, Yang Gao, Pu Chen, Chenyan Lv, Guanghua Zhao

**Affiliations:** 1 College of Food Science & Nutritional Engineering, China Agricultural University, Beijing 100083, China; 2 China Astronaut Research and Training Center, Beijing 100094, China

**Keywords:** wheat protein, gluten network, noodles, enzymes

## Abstract

Upon hydrating and mixing wheat flour, wheat protein forms a network that strongly affects the structure and physicochemical properties of dough, thus affecting the properties of noodles. Different approaches have been taken to alter the gluten network structure in order to control the dough properties. In the current review, we summarize the structure and function of wheat protein, including glutenin and gliadin, and describe food components that may affect noodle quality by interacting with wheat protein. In fact, the ratio of glutenin to gliadin is closely related to the viscosity of dough, and disulfide bonds also contribute to the gluten network formation. Meanwhile, wheat protein coexists with starch and sugar in wheat dough, and thus the nature of starch may highly influence gluten formation as well. Salts, alkali, enzymes and powdered plant food can be added during dough processing to regulate the extensional properties of wheat noodles, obtaining noodles of high quality, with improved sensory and storage properties. This review describes specific methods to reinforce the wheat protein network and provides a reference for improving noodle quality.

## 1. Introduction

The main traditional basic meal in our daily diets consists of wheat products. In Asian countries such as China and Japan, wheat flour is largely utilized for noodles production [1]. Generally, rheological and textural properties such as chewiness and compactness, sensory properties such as aroma and color, and storage properties highly affect noodles acceptance by consumers [2]. Wheat flour has many components such as starch, protein, lipid, and enzymes. The gluten protein forms the principle network of dough, endowing it with elastic, cohesive and viscous properties [3]. It has been reported that gluten quality is highly correlated with noodle texture such as springiness and the cooking loss of noodles [4]. Gliadins and glutenins are the main components of gluten protein. During dough development, gliadins contribute to the viscosity of dough, while glutenins are related to its elasticity [5].

According to previous studies, A-type starch with a disk or lenticular shape was able to make the gluten network more porous and less compact [6]. Other food components in noodles such as salt, alkali and polyphenols were reported to affect the non-covalent and covalent interactions of gluten protein, thereby influencing the physicochemical, sensory and storage properties of noodles [7].

This review focuses on “wheat protein”, “interactions”, and “noodle quality”. We searched these keywords in the “Web of Science” database, filtering irrelevant literature based on article abstracts. After that, we analyzed the selected 85 references. The purpose of this review is to provide a thorough summary of the structure and function of important proteins in wheat and the interactions between wheat proteins and other food components affecting noodle quality. The textural, sensory, and storage qualities of noodles in the food industry will be improved as a result of this review.

## 2. Structure and Function of Wheat Protein

**Figure 1:**
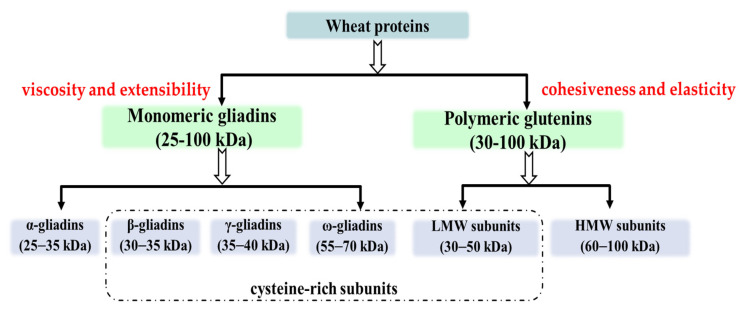
Classifications of wheat protein.

As previously noted, gluten makes up approximately 80% of the protein in wheat flour. According to the solubility of gluten protein in an ethanol–water solution, gluten protein can be divided into two fractions: the soluble fraction contains gliadin, and the insoluble fraction contains glutenin [8]. The glutenin and gliadin proteins have diverse functions during dough production due to differences in their structural compositions (Figure 1). Glutenins form polymers stabilized by inter-chain disulfide bonds, whereas gliadins are monomers and interact with glutenin polymers through non-covalent forces, especially hydrogen bonds [9]. It is commonly believed that glutenin proteins create the polymeric protein network that gives dough its cohesiveness and elasticity, whereas gliadins as plasticizers of the glutenin network and provide dough with viscosity and extensibility [9,10]. Although there are several reviews on high-molecular-weight glutenin, a comprehensive review on the structure and function of gluten protein is lacking.

### 2.1. Glutenin

**Figure 2:**
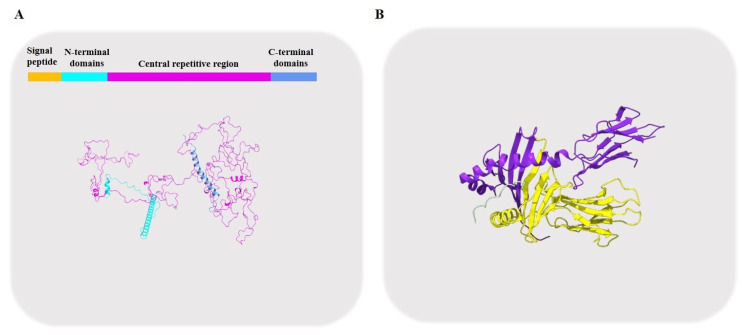
Structure of glutenins. (**A**) Predicted structure of HMW-GS; (**B**) Crystal structure of LMW-GS (PDB: 6px6). The yellow and purple colors show two subunits of LMW-GS.

Glutenin, one of the largest polymers found in nature, is composed of huge macropolymers containing high-molecular-weight (HMW-GS) (Figure 2A) and low-molecular-weight (LMW-GS) subunits (Figure 2B) crosslinked via intermolecular or intramolecular disulfide bonds. According to their electrophoresis mobility in SDS-PAGE, the subunit masses of HMW-GS are between 60,000 Da and 100,000 Da, while those of LMW-GS range from 30,000 to 50,000 Da, and they account for about 20% and 80% of the total glutenin fraction, respectively [11]. HMW-GS mostly influence the final quality of dough.

#### 2.1.1. High-Molecular-Weight Glutenin Subunits

Genes at the *Glu-A1*, *Glu-B1* and *Glu-D1* loci on chromosomes 1A, 1B and 1D code for the HMW subunits. Each low-molecular-weight x-type subunit and high-molecular-weight y-type subunit are encoded by two genes in each locus [12]. Theoretically, hexaploidy wheat should contain six expressed HMW glutenin subunits. In fact, due to gene silencing and allelic variation, the composition of HMW-GS often varies among wheat cultivars [13]. Generally, all cultivars contain 1Bx, 1Dx and 1Dy subunits, while some cultivars also contain a 1By and/or a 1Ax subunit. Therefore, specific composition of HMW glutenin subunits have been identified as standards for wheat cultivar selection.

Four regions form the fundamental structure of an HMW glutenin: the signal peptide (cleaved after maturation), the N- and C-terminal domains, and a central repetitive region (Figure 2A). Cysteine residues that are highly conserved in terms of both number and position can be found in the N- and C-terminal regions of these proteins. The N-terminal domain has between 81 and 104 residues, whereas the C-terminal domain has 42 residues. [12]. Repeats encoding tri-, hexa- and nonapeptides are the primary components of the repetitive domain. In x-type subunits, the repeat units are hexapeptides (PGQGQQ) and nonapeptides (GYYPTSLQQ), while hexapeptides (PGQGQQ) and nonapeptides (GYYPTSLQQ) make up the repeat units in y-type subunits [14]. Differences in subunit size are primarily due to variations in the quantity of tripeptides and hexapeptides. The HMW-GS function is molecularly based on these domains.

The higher order structure of the HMW glutenin subunits is maintained by interactions involving the conserved cysteine residues and the repetitive domain, which mainly include disulfide bonds and hydrogen bonds, respectively [15]. More importantly, because interchain disulfide links favor the production of gluten aggregates and play a significant role in stabilizing HMW-GS polymers, the cysteines in HMW-GS are particularly critical to the structure and function of gluten [16].

The repetitive structure of HMW-GS has been studied by scanning tunnelling microscopy, showing that reverse β-turns and β-sheet are organized in a β-spiral structure, whereas the non-repetitive N- and C-terminal domains are rich in α-helixes [17]. It has been reported that the highest the content of β-turns, the greatest wheat dough viscoelasticity and that HMW-GS with the largest amount of β-sheets produced the strongest wheat dough [18]. However, the crystal structure of HMW-GS is lacking.

**Figure 3:**
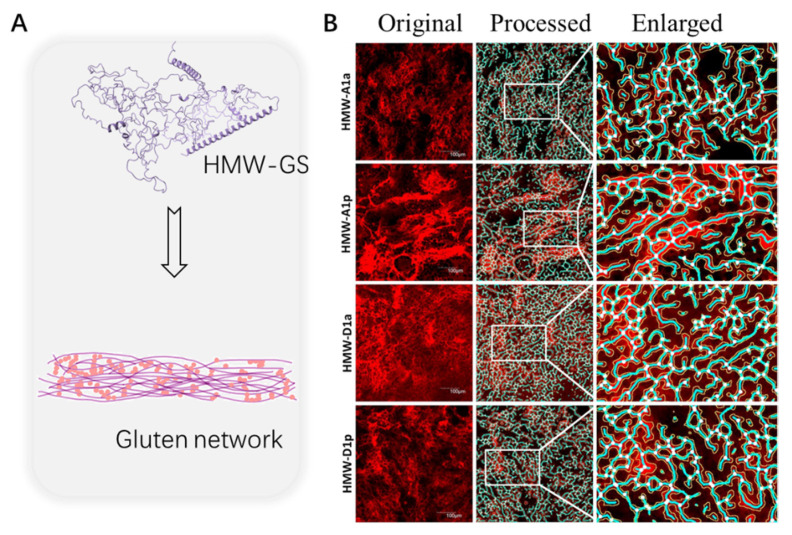
Role of HMW-GS in the gluten network (**A**) and confocal laser scanning microscopy of gluten samples of four wheat lines (**B**). Adapted with permission from Ref. [22]. 2018, Elsevier.

As for the HMW-GS variations in different species, two novel HMW-GS as 1Dx2^s^ and 1Dx2^f^ in the wheat line CNU608 were identified; the introgression of these subunits is closely related to the improvement in dough strength. Specifically, the 1Dx2^f^ subunit includes an additional cysteine residue at position 730 and has a longer repetitive domain and a greater glutamine content, whereas the 1Dx2^s^ subunit presents an octapeptide deletion in the N-terminal region. All these factors help create dough of high quality (Figure 3) [19]. Meanwhile, HMW-GS are closely connected to the dough final quality. Jiang et al. found that HMW-GS affect loaf volume and crumb structure significantly, and their lack may lead to a decline in the size, brightness and fineness of the bread crumb [20]. According to Song et al., gluten quality is weakened when Dx2 is absent from the Glu-D1 locus; genes encoding Glu-1Dx2+1Dy12 were down-regulated in HMW-D1a during grain development, which could affect the glutenin macropolymer [21]. Further research on HMW-GS during food processing is highly needed.

#### 2.1.2. Low-Molecular-Weight Glutenin Subunits

As previously mentioned, low-molecular-weight glutenin subunits (LMW-GS) endow dough with unique viscoelastic properties that enable flour to be processed into a variety of foods [23]. In wheat grain, LMW-GS include around 60% of glutenins and 40% of the total storage protein. In contrast to HMW-GS, the characterization of LMW-GS is difficult, mainly because of the mutigenic of LMW-GS and their low solubility after reduction of the intermolecular disulfide bonds. In general, a signal peptide of 20 amino acids (cleaved after maturation), a small N-terminal domain of 13 amino acids, a repetitive region of variable length, and a C-terminal domain composed of C-terminal I, C-terminal II, and C-terminal III are present in LMW-GSs [24]. According to the first amino acid residue of the N-terminal domain, LMW-GSs are classified into three groups, i.e., LMW-s (serine), LMW-m (methionine) and LMW-i (isoleucine). All genotypes examined have the highest abundance of LMW-s-type subunits, which have an average molecular mass of 35,000–45,000, higher than that of LMW-m-type subunits (30,000–40,000). As for the N-terminal amino acid sequence of LMW-s-type subunits, its sequence is SHIPGL-. In contrast, the N-terminal sequences of the LMW-m type subunits differ dramatically, being METSHIGPL-, METSRIPGL- and METSCIPGL- [25].

LMW-GS was first identified by gel filtration of extracts of wheat flour, distinguishing them from monomeric gliadins. According to their mobility in SDS-PAGE, glutenin subunits can be subdivided into A (HMW-GS), B and C groups (LMW-GS) [26]. Based on their structural characteristics, the B-type subunits extend the growing polymers by forming two intermolecular disulfide bonds, while the C-type subunits serve as chain terminators of the elongating polymer by forming an intermolecular disulfide bond with just one cysteine [26,27].

More recently, new classes of low-molecular-mass proteins have been discovered. Anderson identified a new wheat endosperm protein with distinctive N-terminal sequences, a much smaller central repetitive domain, and much more cysteine residues [28]. Ikeda et al. [29] were able to develop specific PCR and 2DE to distinguish 12 groups of LMW-GS genes in Norin 61. The largest LMW-GS was identified from Aegilops *uniaristata* and is known as LMW-N13. It also has an extra cysteine residue. Transgenic wheat overexpressing LMW-N13 has demonstrated enhanced dough properties. In the meantime, the complete identification of LMW-GSs encoded by Glu-3 loci alleles was made possible through the use of capillary electrophoresis and RP-HPLC. The best quality parameters were found in wheat varieties with the Glu-3 loci scheme [30].

Much more recently, research on celiac disease, which is caused by an immune response to cereal gluten proteins, has resolved the crystal structure of low-molecular-weight glutenin subunits [31]. Glut-L1 (PFSEQEQPV) was reported to bind to celiac disease patient-derived TCR, differently from that of gliadin (Figure 2B). Further characterization of LMW-GS is highly needed to elucidate their role in the formation of the gluten network and in human health.

### 2.2. Gliadin

Gliadins are monomeric proteins and confer foaming and viscous properties on dough. Gliadin is a single-chained polypeptide composed of five amino acids, soluble in 70% ethanol, with molecular weights ranging from 25 to 100 kDa. According to their electrophoretic mobility and genetic data, gliadins can be classified as α (25–35 kDa), β (30–35 kDa), γ (35–40 kDa) and ω (55–70 kDa) (Table 1) [32]. Generally, the gliadin structure contains two primary domains: a hydrophobic center region, which is rich in glutamine and proline, and a terminal hydrophobic portion, which surrounds the central hydrophobic area and is rich in hydrophobic amino acids. Gliadin has a generally limited water solubility, which increases at extremely low pH conditions. The poor aqueous solubility could be caused by its stable disulfide bonds and hydrophobic interactions [33]. Thus, various extraction methods have been used to isolate gliadin. Sardari et al. [34] tried to sequentially use different solvents, such as NaCl, ethanol and an alkaline solution, to isolate the albumin, globulin and prolamin fractions. The isolation and quantitation of gliadin fractions are not reviewed in detail here, and we refer the reader to Mehanna et al. [35]. Importantly, it has been proved that the proportions of gliadin and glutenin can affect the functionality and rheological characteristics of wheat protein. Gliadin transforms into a viscous liquid after being hydrated, providing dough with extensibility and viscosity [36].

## 3. Food Components Affecting the Protein Network and the Properties of Noodles

**Figure 4:**
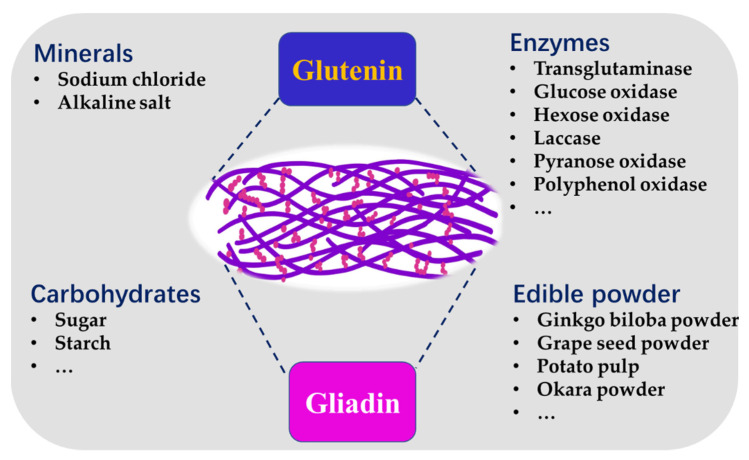
Food components affecting the gluten network of noodles formed by glutenin and gliadin.

The physicochemical, sensory and storage properties of noodles are highly affected by the constituents of wheat flour. In addition to wheat protein, the minerals, carbohydrates and enzymes present in wheat flour can affect the quality of noodles. Recently, with the development of food science and technology, researchers have tried to improve the properties of noodles by adding other food components. In this section, we will summarize the food components added during the production of noodles and their interactions with the wheat protein network (Figure 4).

### 3.1. Minerals

#### 3.1.1. Sodium Chloride

In addition to wheat flour and water, salt is also an important ingredient in noodle processing. Sodium chloride and alkaline salts (e.g., sodium and potassium carbonate, sodium hydroxide and bicarbonates) are commonly used to enhance noodle flavor and texture. In noodle processing, salts exert the following functions. First, salts enhance the flavor and improve the texture; second, they inhibit enzyme activities and microorganisms growth; third, they strengthen and tighten the gluten network of dough [37]. NaCl can strengthen the dough because it can influence protein hydrophobic and electrostatic interactions, thereby impacting the aggregation and disaggregation of proteins. Additionally, NaCl enhances the association of gluten proteins and the rheological properties of noodles. The hardness of machine-made noodles can be increased by adding NaCl (1–2%), improving their quality [38]. On the contrary, during the production of Chinese traditional hand-stretched dried noodle (CHDN), more salt (>3% *w*/*w*) must be used to improve the extensional properties of CHDN dough. According to a study, adding NaCl (1–4% *w*/*w*) improved the dough storage modulus (G′), the loss modulus (G″), the extensional area and the (maximum) resistance to extension. G′ and G″ reflect changes in elasticity and viscosity of wheat gluten protein, respectively. However, an excessive aggregation of protein after adding 5–6% of salt, leads to an opposite result. Therefore, a concentration of salt around 3–5% (*w*/*w*) is ideal to produce CHDN [39]. Chen et al. [40] reported that NaCl increases the mixing time, stability, storage and loss modulus of wheat flour doughs. In addition, salt addition may promote the formation of β-sheets and alter the secondary structure of the protein. Meanwhile, the reduction of the free SH content of gluten indicated that salt induces SH cross-linking during dough production.

#### 3.1.2. Alkaline Salts

The G′ and G″ of gluten and glutenin components are also increased by adding alkali. Alkali addition significantly enhances gluten strength and noodle texture. Gluten strength can differ depending on the physical and chemical characteristics of glutenin and gliadin. Alkali enhance alkali/protein–protein interactions and electrostatic interactions in gluten, which involve, respectively, glutenin and gliadin [41]. *Kan Sui* is the most common alkaline salt used today and contains Na_2_CO_3_ and K_2_CO_3_ or a combination of the two. With the addition of alkaline reagents, the noodle dough becomes tougher, tighter and less extensible [37]. The effect of alkali salts on noodle dough depends on salt concentration. Alkali salts not only can give an elastic mouthfeel to noodles, but also can enhance dough elasticity and strength, making noodles harder and more difficult to break. This may be due to the fact that, in the presence of *Kan Sui*, additional polymeric glutenin can be integrated into the network by thiol (SH)/disulfide (SS) exchange or other non-redox reactions/interactions [42]. The addition of both salt and *Kan Sui* during the sheeting process can significantly increase the noodle dough’s rupture stress. At the same time, the number of β-sheets in the dough is also increased, which makes the structure of the gluten network more stable and delays its disintegration. Furthermore, *Kan Sui* at a low concentration (1%) can significantly inhibit glutenin macropolymer (GMP) dissociation by facilitating disulfide cross-linking and thus enhances the dough’s resistance to stretching [7]. Additionally, Wang et al. [43] reported that phosphate salts increase the rigidity and elasticity of noodles by changing the behavior of the gluten proteins. The specific interactions between gluten proteins and salts need to be further investigated, especially the structure of the complexes they form.

### 3.2. Carbohydrates

#### 3.2.1. Starch

The carbohydrates in wheat flour include starch, dextrin, cellulose, free sugars. The proportion of starch is quite high, at 70–80%. The gelatinization process of starch can alter the textural quality, sensory evaluation and cooking quality of noodles. The primary components of starch in wheat flour are amylose and amylopectin, and the content of amylopectin is higher than that of amylose [2]. However, the amylose content has been reported to positively correlate with noodles’ hardness, gumminess and chewiness, and negatively correlate with their cohesiveness, springiness, and resilience. When the protein content was kept constant, the hardness of noodles was significantly elevated with increasing amylose content. It is possible that the amylose chains leach out of the granules during gelatinization and establish interactions and junctions during noodle cooking. In addition, high smoothness and low adhesiveness are highly desired in noodles [44,45].

Protein and starch interactions have a significant impact on the pasting and textural qualities of starch as well as on the flow, structure and mouthfeel of food products. The elasticity of noodles was adversely associated with protein and amylose content and retrogradation. During heating and cooling, it was discovered that protein had an impact on the peak values of starch’s storage (G′) and loss (G″) moduli [46]. The interactions between starch and protein are important determinants of noodle quality. It was reported that the starch–protein matrix consists of two phases: a dispersed phase that contains starch particles and protein, and a continuous phase that contains amylose/amylopectin [47]. In addition, the alkyl side chains of proteins contain many hydrophilic groups (amides, hydroxyl, carboxyl and mercaptan) that could interact with the starch molecules [48]. Furthermore, the starch–protein interactions are determined by a combination of different forces, such as covalent bonds, electrostatic forces, van der Waals forces, hydrogen bonds and hydrophobic interactions [49]. On the other hand, as the temperature decreases, starch retrogradation occurs, during which the side chains of amylose and amylopectin molecules are rearranged and recombined. With the help of gluten, the free hydroxyl groups of starch form hydrogen bonds with free amino acid residues, delaying the regeneration of starch. Additionally, gluten forms a double helix with amylose in the starch–protein paste system, which prevents starch’s short-term retrogradation [50]. These interactions between starch and protein affect the quality of the noodles. Further research is needed in the future to better understand how proteins alter starch conversion.

Damage to starch occurs when wheat flour undergoes mechanical damage caused by external processing conditions. Because the internal structure of granules becomes exposed following damage, water absorption by starch and its sensitivity to enzymes increase. This means that, compared to intact starch, damaged starch can more easily absorb water and swell, whereas intact starch is protected by a physical barrier that prevents the gluten network from growing [51]. As damage increases in starch, the springiness and hardness of noodles are significantly enhanced. It is well known that damaged starch has a higher water absorption capability than intact starch. Hence, high levels of damaged starch disrupt the integrity of the gluten protein network and decrease the development of a raw noodle dough by competing with proteins for water [45,52].

The swelling power of starch is closely related to noodle eating quality. According to Yue et al. [53], cooking loss is inversely correlated with flour swelling power, indicating that starch with a higher swelling power might be properly incorporated into the gluten network structure, reducing the flour swelling power, which would then decrease the cooking loss. Furthermore, frozen cooked noodles (FCNs) can additionally be broken by ice crystals; however, the interactions of starch with proteins can decrease the damage. Previous research revealed that the content of damaged starch was positively correlated with the expansion capability of wheat flour as well as the hardness, chewiness, and elasticity of the resulting FCNs. However, with an increase in the amount of damaged starch, the gluten network structure is destroyed [54].

Hydrogen bonds are broken and rearranged during the starch gelatinization process. The combined action of heat and moisture gelatinizes starch when fresh noodles are cooked. Then, as moisture slowly permeates the noodles from the outside to the inside, the gluten protein thermally polymerizes, creating a compact three-dimensional network structure embedded in the starch. Finally, a layer with a high moisture level on the outside and a low moisture content within develops, providing the noodles with good taste and viscoelasticity [55,56]. Previous research has shown that temperature and wheat starch gelatinization have an impact on the color, firmness and viscoelasticity of noodles. During gelatinization, gluten is adsorbed into the starch granules through hydrophobic interactions between gluten and the starch granules, which causes gluten and starch to hydrate in a competitive manner and may prevent water from diffusing into the starch granules [50]. In addition, the physicochemical properties of gluten–starch mixtures can be influenced by different gliadin/glutenin ratios. As the gliadin/glutenin ratio increases, the viscosity decreases. This might be because gliadin has many hydrophilic residues on its surface, which increases the binding of water molecules to gliadin and decreases the viscosity of the gelatinized starch [50].

#### 3.2.2. Sugar

The presence of the sugar sucrose entails a significant modification of the rheological properties of dough. With the addition of sucrose, dough’s stickiness and extensibility are improved, but its consistency and tenacity decrease. It is possible that the affinity of sucrose for water inhibits water absorption by starch and gluten, and the resulting dough protein networks require a longer time to unfold [57]. Furthermore, it would take more energy for the nonpolar side chains of aliphatic and aromatic amino acids to become exposed in a sucrose solution. As a result, for protein cross-linking to occur during processing, the temperature must be raised [58,59].

As a low-molecular-weight polyol and plasticizer, sorbitol has the potential to considerably slow down the degeneration of the gluten network. It can improve the hydrogen bonding connections in the gluten system and then promote the dynamic depolymerization and repolymerization of gluten protein molecules throughout processing and cooking, stabilizing the dough’s texture. In a study, sorbitol was added in an appropriate amount (2%), which increased gluten’s tensile strength and dough’s viscoelasticity. It also reduced the weight of the glutenin macropolymer (GMP), the cooking loss of new noodles and the dough’s hardness and springiness [4]. Although the effects of carbohydrates on the properties of noodles have been extensively investigated, the interactions between carbohydrates and glutenin or gliadin during dough formation and the effects on noodle properties need to be further elucidated.

### 3.3. Enzymes

Since enzymes in wheat flour are fully denatured during processing, exogenous enzymes are widely used to crosslink gluten directly or indirectly and improve dough quality. The creation of covalent connections between polypeptide chains is facilitated by enzymes that catalyze the oxidative crosslinking of SH groups and tyrosine residues or acyl-transfer processes between amino acid residues [60].

#### 3.3.1. Transglutaminase

An acyltransferase known as transglutaminase (TG) is used in several culinary products such baked goods, meat, shellfish and others. For example, TG was reported to improve the elasticity, water capacity and other functional properties of wheat dough through amine incorporation, crosslinking and deamidation [61]. Previous research reported that TG addition was able to affect the wheat flour pasting properties and increase the elastic modulus (G′) and the viscous modulus (G″) in doughs. TG can also help frozen doughs to develop a gluten network and fewer isolated starch granules [62]. Upon the addition of 1 g/kg of TG, the storage modulus and loss modulus of white salted noodle dough increased significantly; however, with a higher concentration of TG, the effects on these parameters were not clear. For dried white salted noodles, the textural parameters generally increased, including tensile force, hardness and gumminess [63]. This result can be explained by the fact that TG can catalyze the acyl transfer reaction between lysine and the γ-carboxamide groups of a peptide-bound glutaminyl residue in proteins, yielding intra- and intermolecular 3-N-(γ-glutamyl)-lysine crosslinks between proteins. Crosslinking strengthens the gluten network, enhancing the strength and elasticity of dough [64]. In addition, HMW-GS and α-gliadins are predominantly involved in cross-links formed by TG. TG has a greater effect on HMW-GS than on gliadins, because of the lower lysine content of gliadins as compared to glutenins. However, a higher dosage of TG improved the formation of a protein network in flour, causing an uneven distribution of wheat protein structures [65,66].

#### 3.3.2. Glucose Oxidase

GOX is a glycoprotein that mostly consists of mannose and has a 16% carbohydrate content. The capacity of glucose oxidase (GOX) to promote cross-linking within the gluten network preserves the texture of noodles when frozen. When oxygen is present, GOX catalyzes the oxidation of α-D-glucose to α-D-gluconolactone and H_2_O_2_ in wheat flour. It is believed that H_2_O_2_ is the catalyst for GOX in dough and that the free thiols in cysteine, peptides, and proteins in dough are potential reaction sites. For example, H_2_O_2_ can form disulfide bonds and bind the ferulic acid residues of arabinoxylan into the gluten structures by reacting with free thiol groups in glutenin proteins, thus improving gluten quality and enhancing dough’s extensibility [67,68]. Bonet et al. reported that GOX modified gliadin and glutenin by the formation of disulfide and non-disulfide crosslinks. With the addition of GOX, a reinforcement or strengthening of wheat dough can be obtained. However, a large amount of GOX in dough leads to excessive gliadin cross-linking and aggregation, as well as to severe water-soluble arabinoxylan gelation, weakening the glutenin network and making dough less extensible [69]. Several research groups found that adding GOX to dough boosts dough’s water absorption, toughness and elasticity but decreases its extensibility [60]. However, the mechanism of GOX action is poorly understood at the molecular level.

The addition of the above-mentioned enzymes to wheat dough improves the physicochemical characteristics, handling properties and shelf-life of noodles. In addition to transglutaminase and glucose oxidase, Table 2 shows many other enzymes that can be used in noodle processing to improve gluten texture and quality (Table 2).

### 3.4. Edible Powder from Plant Sourcse

With the economic development and the increasing consumer awareness of the importance of a healthy lifestyle, it is increasingly important to emphasize that noodles are rich in natural protein and other useful plant-based ingredients. Researchers have attempted to improve the nutritional value and quality of noodles by fortifying them with a variety of ingredients, over the past few years [73].

The quality of dough and fresh wet noodles can be significantly enhanced by ginkgo biloba powder (GBP). A study found that the mixture tolerance index of dough, its water absorption, G′ (storage modulus) and G″ (loss modulus), extensibility, hardness, adhesiveness, springiness and chewability increased with the addition of GBP. However, dough viscosity, setback, breakdown, development time dough stability decreased. The addition of GBP in an amount less than 20% is appropriate [74]. With an increasing GBP concentration in a GBP/wheat flour mixture, the amount of moisture, protein, total sugar, starch and gluten progressively dropped, whereas the content of flavonoid, fiber and amylose increased. The great number of hydroxyl groups existing in fiber structure allow more interactions with water through hydrogen bonding, resulting in a better water absorption by gluten; additionally, such interactions change the way of water distribution inside the dough and how the gluten networks form [75]. Meanwhile, with a lower gluten content in the dough, it usually takes a longer time to build the gluten network, and the starch–gluten interactions are less stable [76].

**Figure 5:**
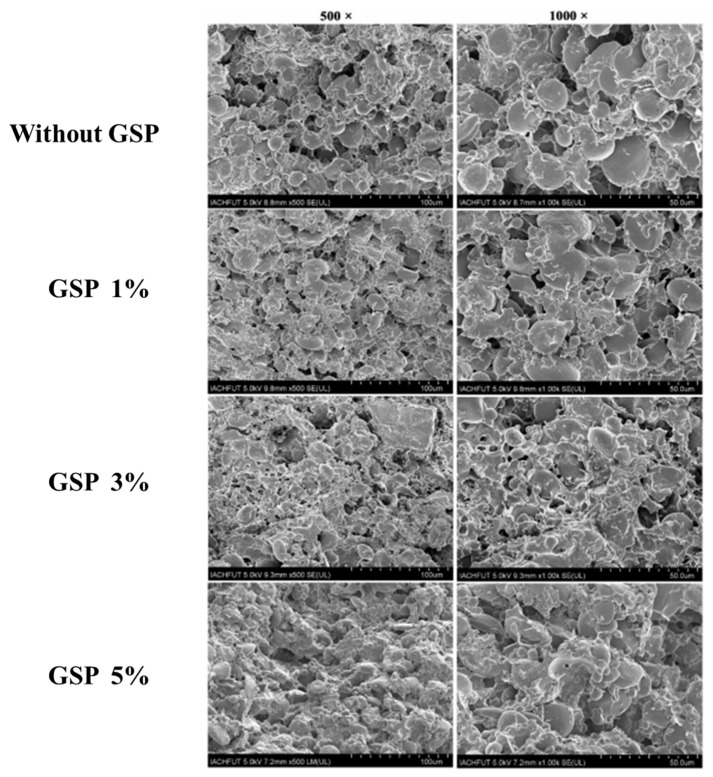
Role of grape seed power in the structural properties of the gluten network of noodles. Adapted with permission from ref. [1]. 2021, Elsevier.

Grape seed power (GSP) has been used in some food products because it may have health advantages. The hardness, adhesiveness and chewiness of noodles increased initially with an increase in the GSP concentration and then decreased; recovery and adhesiveness essentially remained the same, and elasticity declined. It is reported that moderate amounts (1%) of GSP significantly improved the quality of raw noodles [77], which indicated that the addition of GSP broke the disulfide bonds in gluten proteins, since the amount of free sulfhydryl groups in gluten proteins increased, and the hydrophobic region on the surface shrank. However, both hydrogen bonding and hydrophobic interactions helped the gluten proteins aggregate. Meanwhile, little amounts of GSP active constituents may interact with the proteins in gluten to create a tighter network structure (Figure 5), raising the level of quality of the noodles. On the contrary, an excessive GSP addition will lead to more sulfhydryl groups and less disulfide bonds in the structure of gluten, hindering the establishment of the gluten network and favoring its destruction [1].

Potato pulp addition may affect the processing properties and gluten structure of wheat dough. The development time, water absorption, storage modulus (G′) and loss modulus (G″) of dough were negatively impacted by the amount of potato pulp. The stability and extensional characteristics of the dough decreased in the presence of a high potato pulp content (≥30%), which also influenced the dough’s processing properties. The gelatinization characteristics also revealed that wheat flour containing potato pulp had decreased viscosity. Meanwhile, the content of free SH in wheat flour containing 30–50% potato pulp increased, and the content of S-S decreased, which significantly decreased the number of β-sheet and α-helix structures in the dough [78]. This occurred most likely because the dietary fiber in potato pulp may prevent the swelling of the starch granules, which would reduce friction and lessen the viscosity. The disulfide bond content and the β-turn content are connected, but high potato pulp levels will reduce the gluten content and the disulfide bond content [79].

The addition of dried okara powder has been reported to improve the cooking loss, tensile property, elasticity and sensory acceptability of noodles. It was shown that with the addition of optimal amounts of wheat gluten flour (WGF) (2.5–4.7%) and unripe banana flour (UBF) (22–22.5%), white salted noodles with excellent sensory and physicochemical properties could be prepared. The thermal stability, peak viscosity and final viscosity of reconstituted flour can be enhanced by an increased amount of UBF along with WGF, thus improving the overall cooking quality of noodles [80]. According to Sun et al. [3], adding Chinese yam powder (CYP) to wheat flour could influence the general characteristics of dough and noodles. It might promote the tight association of starch particles with gluten protein, leading to a smooth surface. Besides, the addition of 20% CYP increased the product’s hardness, mouthfeel and springiness. The addition of potato flour increased the adhesiveness, hardness, springiness and water absorption of noodles. However, this process destroyed the gluten network structure of the dough, decreased its elastic and viscous modulus, and increased the rake of the noodles when they broke [81]. In addition to Shiitake mushroom powder [82], dragon fruit peel powder [83], oats [84] and buckwheat [85] could affect the structure of starch or protein in noodles and improve their quality. Considering the possibility of improving the quality of noodles with powdered ingredients, the specific ingredients that may affect the protein structure in dough should be identified, and their mechanisms of action should be uncovered in detail.

## 4. Conclusions

Many efforts have been made to improve the quality of noodles, not only fresh and dried noodles, but also frozen noodles. Wheat proteins, especially glutenin and gliadin, are the most important factors that affect the physiochemical, sensory and stability properties of noodles. In this review, we described the classification and essential structure of wheat protein. Moreover, the interactions between food components such as minerals, carbohydrates, enzymes and edible powder from plant sources were summarized. Hydrogen bonds, hydrophobic interactions and disulfide bonds are responsible for the interaction of wheat protein with food components. Although the literature on gluten proteins is already extensive, it is still necessary to determine the structural features of protein through proteomics or X-ray diffraction during dough processing. In addition, multiple factors may contribute to the gluten network, and it is necessary to construct effective models to guide the improvement of noodle quality. This review highlights the recent developments in this field, which is beneficial for the applications of wheat protein in the future. Further studies are encouraged to determine the effects of food components with different particle sizes or proteins with various molecular weights on the nutritional value and quality of noodles.

## Figures and Tables

**Table 1:** Predicted structure of gliadins.

Type	Predicted Structure
α/β-gliadins	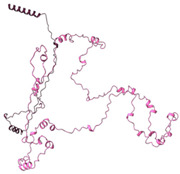
γ-gliadins	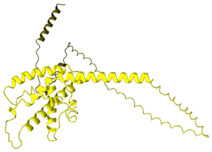
ω-gliadins	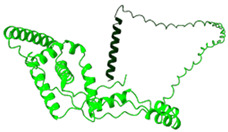

**Table 2:** Enzymes widely used in wheat products.

Enzymes	Effects	References
Hexose oxidase (HOX)	Due to the formation of hydrogen peroxide, HOX causes S-S linkages to form between proteins and boosts the gelation of arabinoxylans. This increases the strength of dough and the volume of bread.	[60]
Laccase (LAC)	LAC treatment reduces dough extensibility and promotes dough stiffness.	[67]
Pyranose oxidase (P_2_O)	The molecular mechanism of improving dough stability by P_2_O involves the cross-linking of gluten proteins and arabinoxylan with formation of H_2_O_2_.	[70]
Polyphenol oxidase (PPO)	PPO would cause discoloration of the noodles.	[71]
Cyclodextrin Glycosyl transferase (CGT)	CGT decreases dough consistency but provides a good specific volume and a very soft crumb texture to bread.	[72]

## Data Availability

Not applicable.
